# Preparative microdroplet synthesis of carboxylic acids from aerobic oxidation of aldehydes†
†Electronic supplementary information (ESI) available. See DOI: 10.1039/c8sc01580e


**DOI:** 10.1039/c8sc01580e

**Published:** 2018-05-16

**Authors:** Xin Yan, Yin-Hung Lai, Richard N. Zare

**Affiliations:** a Department of Chemistry , Stanford University , Stanford , CA 94305-5080 , USA . Email: rnz@stanford.edu

## Abstract

Single liquid-phase and liquid–liquid phase reactions in microdroplets have shown much faster kinetics than that in the bulk phase. This work extends the scope of microdroplet reactions to gas–liquid reactions and achieves preparative synthesis. We report highly efficient aerobic oxidation of aldehydes to carboxylic acids in microdroplets. Molecular oxygen plays two roles: (1) as the sheath gas to shear the aldehyde solution into microdroplets, and (2) as the sole oxidant. The dramatic increase of the surface-area-to-volume ratio of microdroplets compared to bulk solution, and the efficient mixing of gas and liquid phases using spray nozzles allow effective mass transfer between aldehydes and molecular oxygen. The addition of catalytic nickel(ii) acetate is shown to accelerate further microdroplet reactions of this kind. We show that aliphatic, aromatic, and heterocyclic aldehydes can be oxidized to the corresponding carboxylic acids in a mixture of water and ethanol using the nickel(ii) acetate catalyst, in moderate to excellent yields (62–91%). The microdroplet synthesis is scaled up to make it preparative. For example, aerobic oxidation of 4-*tert*-butylbenzaldehyde to 4-*tert*-butylbenzoic acid was achieved at a rate of 10.5 mg min^–1^ with an isolated product yield of 66%.

## Abstract 2

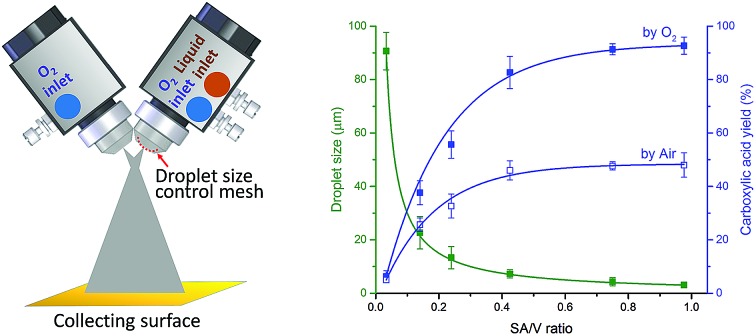
Preparative synthesis of carboxylic acids from the aerobic oxidation of aldehydes in microdroplets with moderate to excellent yields.

## Introduction

Recent findings indicate that reactions in microdroplets created by spray-based ionization/aerosols are extremely attractive, as microdroplet reactions can be many orders of magnitude faster than their conventional bulk-phase counterparts.^1^,^2^ This phenomenon stimulates strong interest in using microdroplets as a chemical synthetic tool. Remarkable acceleration has been observed in single liquid-phase reactions and liquid–liquid phase reactions. For example, an accelerated Pomeranz–Fritsch synthesis of isoquinoline in methanolic solution was detected by on-line mass spectrometry (MS) in charged droplets generated by electrospray.^3^ The rate of this droplet reaction was reported to be 10^6^ times faster than that in the bulk. Liquid–liquid bulk-phase Stevens oxidation of alcohols to the corresponding aldehydes/ketones is inhibited if no phase-transfer-catalyst is used, because of the inability of reagents to come together. In sharp contrast, non-catalytic oxidation can be achieved in microdroplets formed by dual sonic sprays within milliseconds in moderate to good yields.^4^ Other reactions involving C–C,^5^–^7^ C–N,^6^,^8^–^10^ and C–O^11^ bond formation have been reported to be accelerated by factors of 10 to 10^6^ in single liquid-phase solutions.

Gas–liquid reactions are of great chemical, biological, physiological, and ecological importance.^12^,^13^ An important question is whether gas–liquid reactions can be accelerated in microdroplets generated by spray-based ionization methods. Such methods of forming microdroplets often apply a sheath gas (commonly nitrogen gas) to pneumatically assist the formation of the sprayed droplets. An extra advantage of replacing sheath gas with reagent gas will be gained by its dual role as an assistant in droplet formation and as a reagent.

**Fig. 1:**
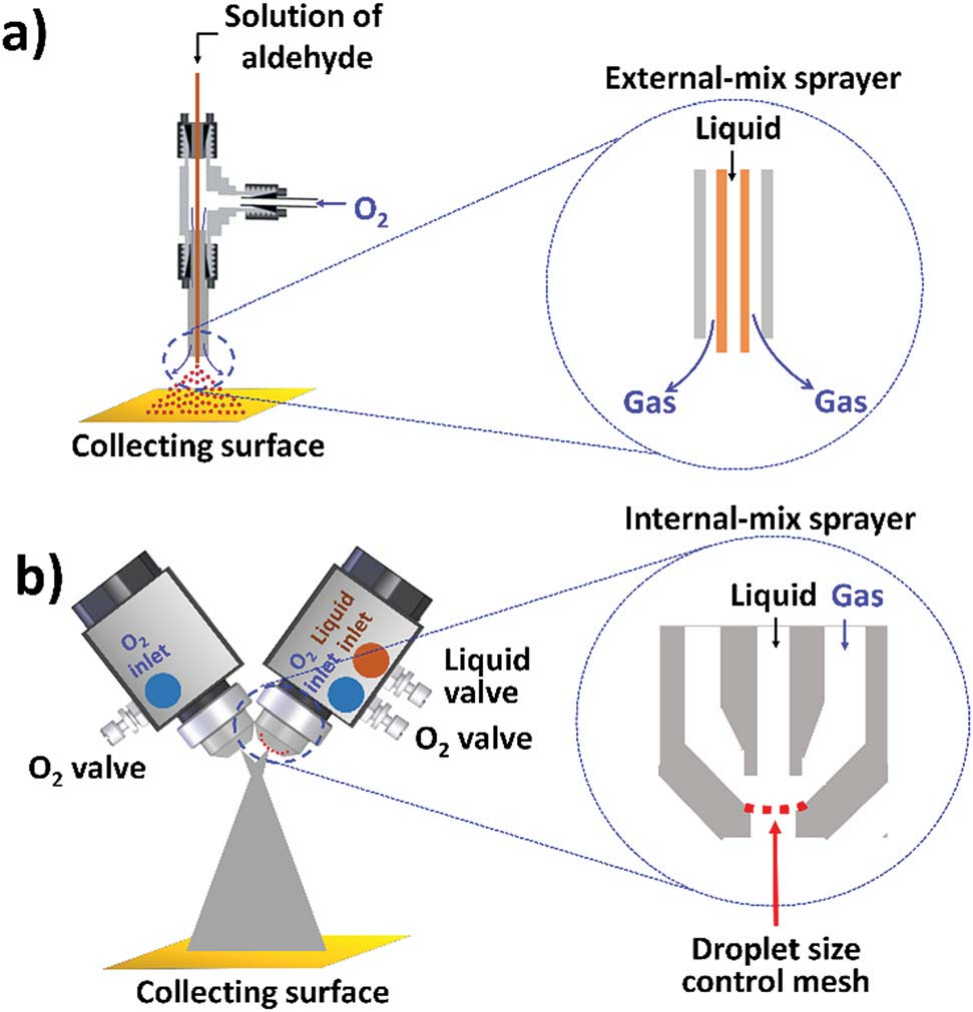
Two-phase aerobic oxidation of aldehyde into carboxylic acid performed in microdroplets: (a) small scale in which the microdroplets are generated by the atomization of the bulk solution with turbulent nebulizing oxygen gas at 90–120 psi (the inset shows the sprayer with external mixing of liquid and gas); and (b) preparative synthesis using modified commercial spray nozzles (the inset shows the nozzle with internal mixing of liquid and gas, and a mounted mesh that controls the droplet size).

The oxidation of aldehydes to carboxylic acids has been of long-standing interest in synthetic organic chemistry,^14^ and is an industrially important process.^15^ Compared to different oxidizing reagents used in conventional methods such as Cr(iv)-based Jones oxidation,^16^,^17^ Ag(i)-based Tollen's reaction,^18^ and Cu(ii)-based Fehling's reaction,^19^ molecular oxygen is considered as an ideal oxidant because it is inexpensive, environmentally friendly,^20^ and exhibits highly atom-efficient oxidation per weight (100% atom efficiency).^21^ Methods to achieve direct and efficient oxidation of aldehydes to carboxylic acids using molecular oxygen as the oxidant under mild conditions are relatively scarce and highly needed,^22^ although recently, progress has been made in the development of less expensive transition-metal catalysts for oxidation of aldehydes to carboxylic acids in the bulk.^22^–^24^ In this work, we report a highly efficient aerobic oxidation of aldehydes to carboxylic acids in microdroplets generated by sonic spray ionization (Fig. 1a). Molecular oxygen plays dual roles of being the oxidant as well as the sheath gas to generate microdroplets. Mixing of two phases occurs during microdroplet formation. The effect of the surface-area-to-volume ratio (SA/V ratio) of microdroplets on the yield of the reactions is also studied.

Another question well worth investigation is the scale of microdroplet reactions, as it determines the practicality for chemical synthesis. Previous studies on “preparative electrospray” employed four or eight spray sources at the same time, and products were generated at rates of *ca.* 1.2–1.6 milligrams per minute for Claisen–Schmidt condensations, benzoin condensations, and Stevens oxidations.^4^,^5^ Further scale-up of microdroplet reactions by paralleling more spray sources might not be practical and economical owing to complicated arrangements of splitting gas and liquid, as well as the large demand for duplicated spray sources. Here, we developed a device applying a high flux of liquid droplets colliding with gas molecules while maintaining suitable microdroplet sizes for fast and large-scale microdroplet synthesis (Fig. 1b).

## Results and discussion

**Scheme 1:**
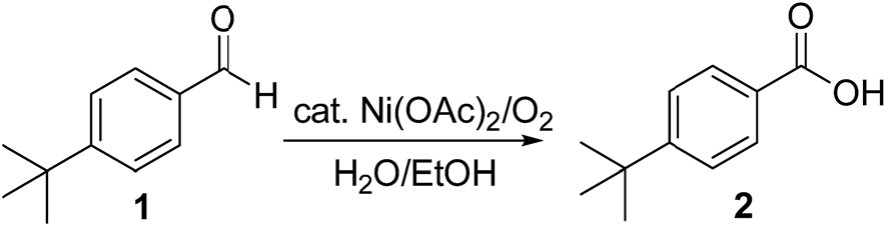
Oxidation of 4-*tert*-butylbenzaldehyde **1** with molecular oxygen and 5 mol% Ni(OAc)_2_ in water–ethanol (v/v = 1 : 1.2) to form 4-*tert*-butylbenzoic acid **2**.

**Fig. 2:**
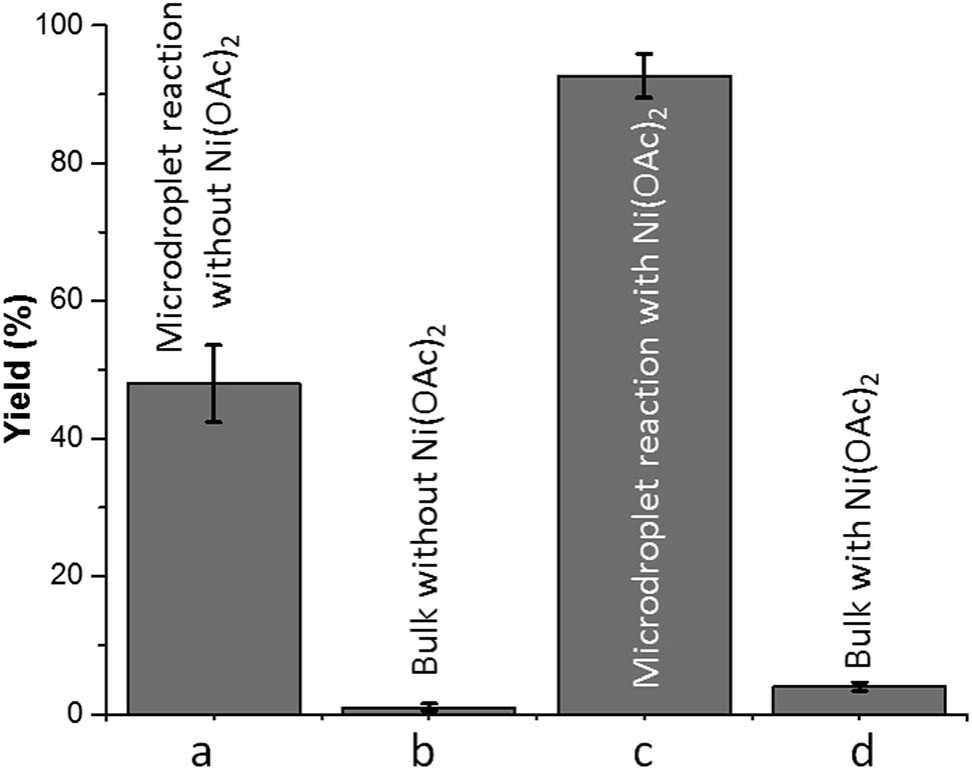
Oxidation of 4-*tert*-butylbenzaldehyde **1** with molecular oxygen in water–ethanol (v/v = 1 : 1.2): (a) in microdroplets without adding Ni(OAc)_2_; (b) in bulk without adding Ni(OAc)_2_; (c) in microdroplets with 5 mol% Ni(OAc)_2_; and (d) in bulk with 5 mol% Ni(OAc)_2_. The collection time for droplet reaction is 30 min and the bulk-phase reaction time is the same. Error bars represent one standard deviation (3 measurements).

We began our investigation by examining (Scheme 1) the oxidation of 4-*tert*-butylbenzaldehyde **1** in a water–ethanol solvent (v/v = 1 : 1.2) with molecular oxygen (O_2_) and without any metal catalyst to form 4-*tert*-butylbenzoic acid **2** at room temperature. A water–ethanol solution of **1** (0.1 M) introduced through a fused silica capillary (i.d. 50 μm) at a rate of 15 μL min^–1^ was atomized into microdroplets (average size *ca.* 3.1 μm; see the ESI† for the method of droplet measurement) with a coaxial flow of oxygen, which was used as the turbulent nebulizing gas operated at 120 psi as well as the sole oxidant. The oxidation of **1** was initiated by the interactions between **1** in microdroplets with molecular oxygen at the interface. The resulting products **2** were collected for 30 min using a microdroplet trapping system (Fig. S1†). The reaction mixture was extracted with dichloromethane and analysed by ^1^H NMR. The yield of **2** from the oxidation of **1** was found to be 48% (Fig. 2a). A control experiment was performed in bulk solution (O_2_ was supplied in a balloon). The reaction mixture was vigorously stirred at room temperature, and less than 1% of the product was detected after 30 min (Fig. 2b). An acceleration factor of 50 was achieved for the reaction in microdroplets compared to the bulk reaction calculated based on the yields obtained after 30 min. Reaction is expected to continue on the surface of the collected film, but the film surface area is so small compared to the surface area of the microdroplets that this contribution to the calculation of the acceleration factor has been neglected.

Next, we screened for possible catalysts without adding any ligand or additive which would promote this reaction in microdroplets, with an emphasis on widely available and inexpensive metal catalysts. Nickel(ii) acetate (5 mol%) showed the best efficiency among all the screened catalysts (Fig. S2†); a yield of 91% was achieved. In contrast, the addition of nickel(ii) acetate improved the yield of bulk reaction to 4% in 30 min (Fig. 2d, ESI† Section 6). We rationalized the observation as follows: the liquid-phase oxidation of organic compounds with O_2_ can be affected by a complex set of factors which include intrinsic parameters (aldehyde reactivity, solvent, *etc.*) and extrinsic parameters (catalyst, initiators/inhibitors, *etc.*), as well as physical phenomena such as gas to liquid mass transfer.^25^ When oxygen transfer becomes the rate limiting step, the rate of the overall process is no longer controlled by chemical mechanisms but rather by physical transport.^25^

**Fig. 3:**
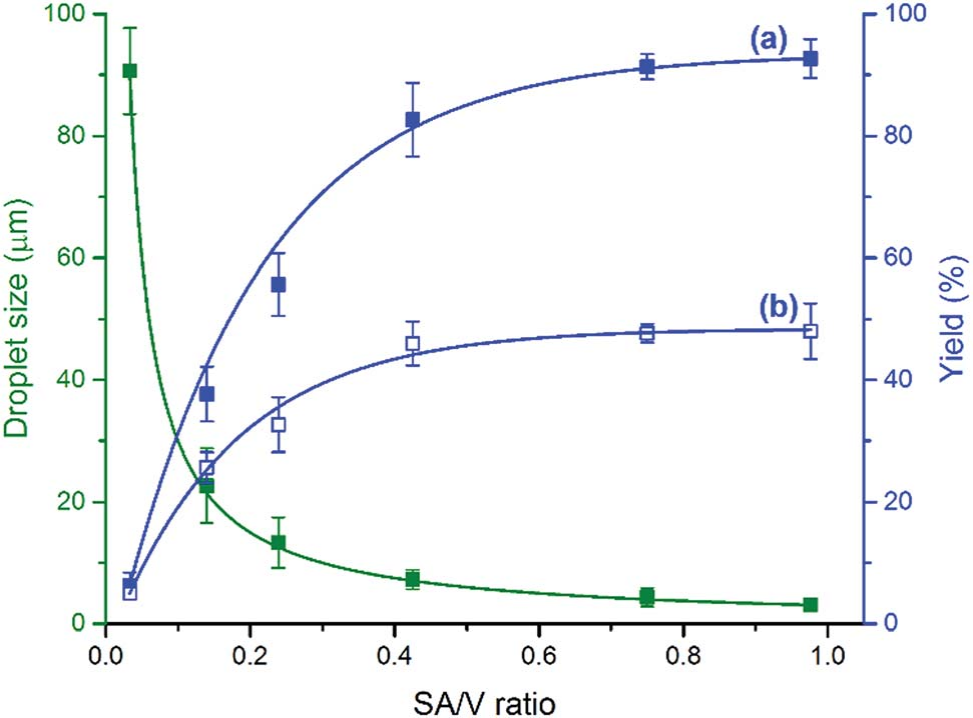
Dependence of product yield on the SA/V ratio in microdroplet aerobic oxidation of 4-*tert*-butylbenzaldehyde to 4-*tert*-butylbenzoic acid: (a) using O_2_ as the oxidant; and (b) using air as the oxidant.

Mass transfer across the interface is the rate-controlling step in most two-phase reactions.^26^ The surface effect has also been observed in atmospheric halogen chemistry,^27^ reactions with Criegee intermediates at the air–aqueous interface,^28^ and catalytic oxidation of *p*-xylene to produce high-purity terephthalic acid,^29^ as well as biphasic reactions in flow systems.^24^,^30^–^33^ These considerations prompted us to investigate the effect of SA/V ratio on the product yield. We controlled the droplet size by varying the pressure of sheath gas and using capillaries with different inner and outer diameters. The SA/V ratio of microdroplets was calculated based on the droplet size measured by micro-particle image velocimetry (μPIV, see the ESI for details†). The experiment started with dripping droplets with a SA/V ratio of 0.002 through the capillary (i.d. 250 μm, o.d. 365 μm) with no sheath gas supply but in an oxygen environment protected by an O_2_ balloon. The flow rate was kept at 15 μL min^–1^, and less than 6% product was formed in 30 min. We increased the SA/V ratio of droplets by up to 500 times by increasing the O_2_ sheath gas pressure from 30 to 120 psi through the capillary (i.d. 50 μm, o.d. 365 μm). The yield of product 4-*tert*-butylbenzoic acid was largely enhanced with an increase of the SA/V ratio of droplets from 0.033 to 1 (Fig. 3a), and reached the maximum yield when the droplet size decreased to about 3 μm. Similar phenomena were also observed using compressed air as the oxidant with less product formation (Fig. 3b). The closely related effect of SA/V ratio on the yield was found for reactions with different aldehydes (Fig. S3†).

The solvent system was investigated, because it not only serves as the reaction medium but also affects the formation of microdroplets.^10^ We found that water–ethanol (v/v = 1 : 1.2) gave the best yields for the droplet reactions among various organic solvents as well as miscible aqueous organic solvents for the microdroplet oxidation of 4-*tert*-butylbenzaldehyde (Fig. S4†).

Encouraged by these results, various aldehydes including aromatic, heterocyclic and aliphatic aldehydes (Table 1) were tested under optimized conditions. The corresponding carboxylic acids were obtained in moderate to good yields (62–91%).

**Table 1:** Aerobic oxidation of various aldehydes in microdroplets

Reagent	Product	Yield (%)
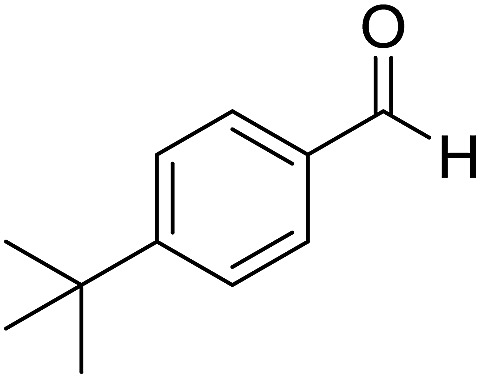	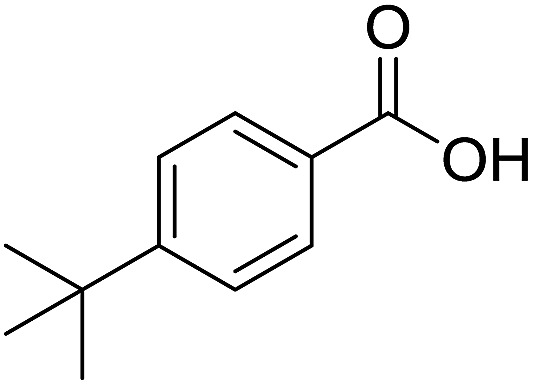	91
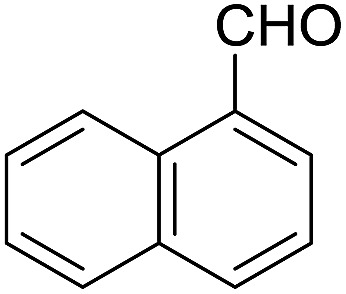	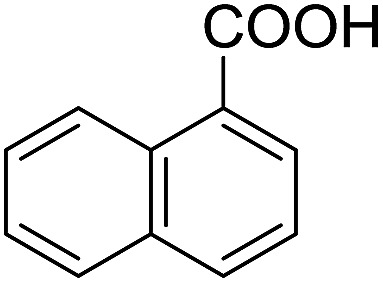	62
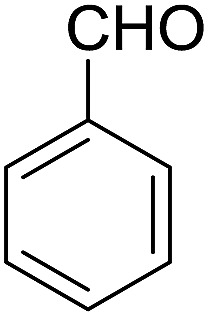	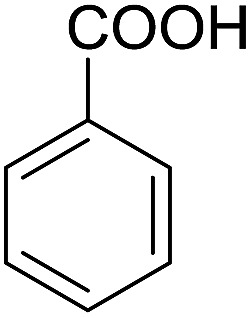	80
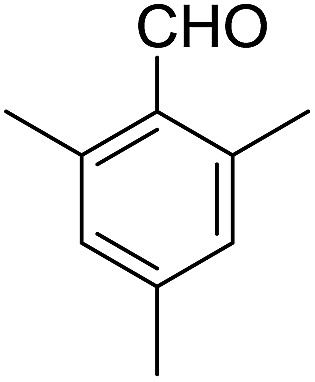	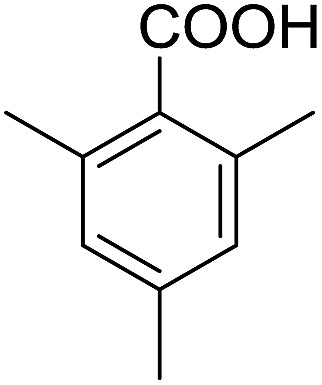	88
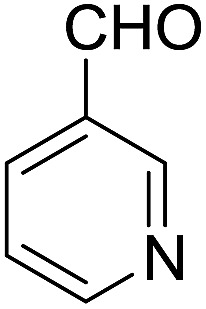	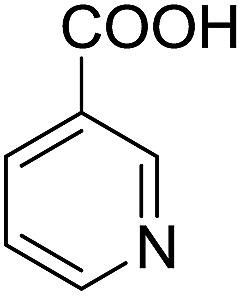	75
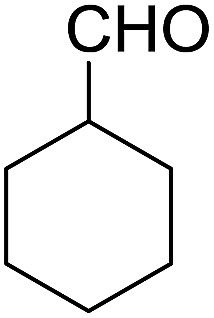	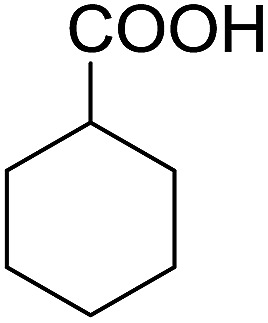	71
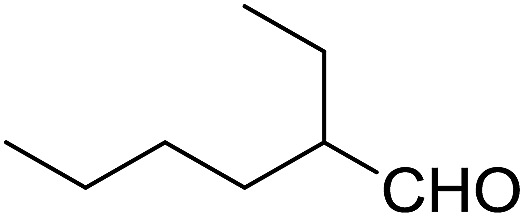	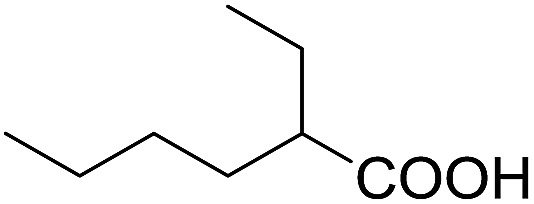	78

The highly efficient transformation of aldehydes into carboxylic acids described above inspired us to explore the possibility of scaling up these reactions in microdroplets. Regular sprayers (electrospray, sonic spray source, *etc.*) applied in previous microdroplet work use concentric capillaries (for liquid reagents) inserted into a sheath gas tubing with a length of 1 mm staying outside (Fig. 1a inset). The sheath gas comes into contact with the liquid outside the sprayer and shears the liquid into microdroplets. Simply enlarging the capillary size and increasing the liquid flow rate from previous spray sources (Fig. S5a†) resulted in incomplete atomization of the liquid (especially for the liquid in the middle of the flow), as well as a large distribution of droplet sizes, causing little product (<1%) to be formed. In our design, an internal-mix nozzle (from Unist Co., Grand Rapids, MI) was used in which the sheath gas comes into contact with the fluid inside the nozzle and disperses it into microdroplets flying throughout the spray hole (Fig. 1b inset). Such a nozzle uses less atomizing gas and generates droplets with a smaller size distribution compared to the previous external mix spray of liquids at the same flow rate. It is also better suited to higher viscosity streams.

The problems with direct use of commercialized internal-mix nozzles for microdroplet reactions are (1) the droplets generated from this nozzle are too large (*ca.* 90 μm) for accelerated microdroplet reactions (Fig. 5), and (2) an increased flow rate (8 mL min^–1^) does not allow 4-*tert*-butylbenzaldehyde to make good contact with the oxidant, leading to a reaction yield of less than 5%.

**Fig. 4:**
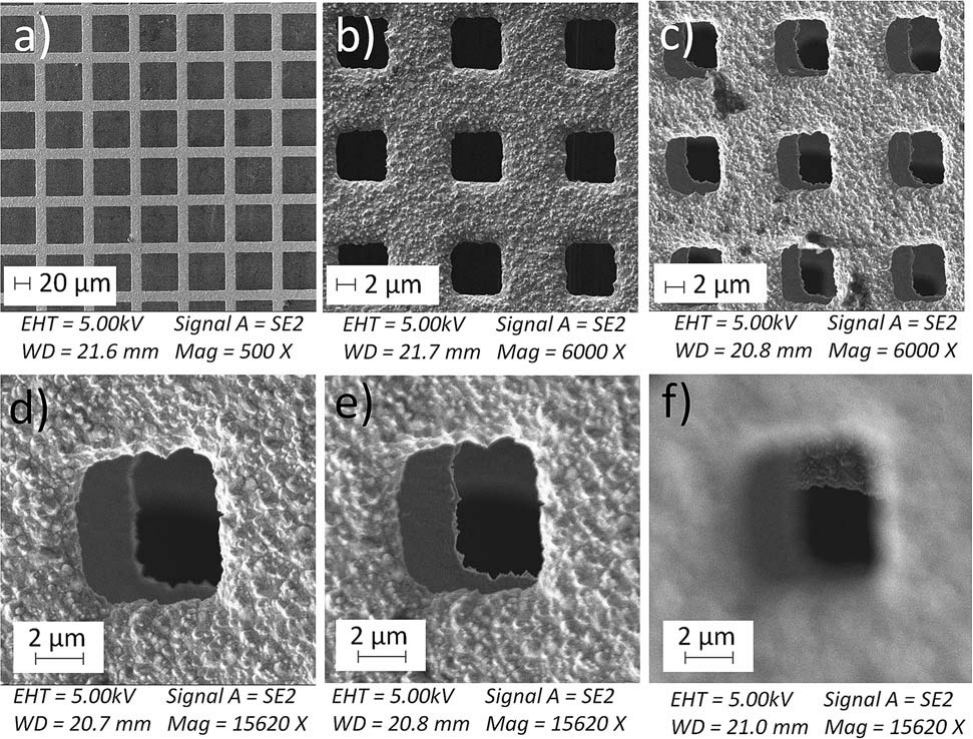
Scanning electron microscopy (SEM) images of meshes with sizes of (a) 50 μm, (b) 5.5 μm, and (c) three layers of 5.5 μm mounted in front of the microdroplet spray nozzle; (d), (e), and (f) show the focus on the first, second and third layer of meshes used in (c).

**Fig. 5:**
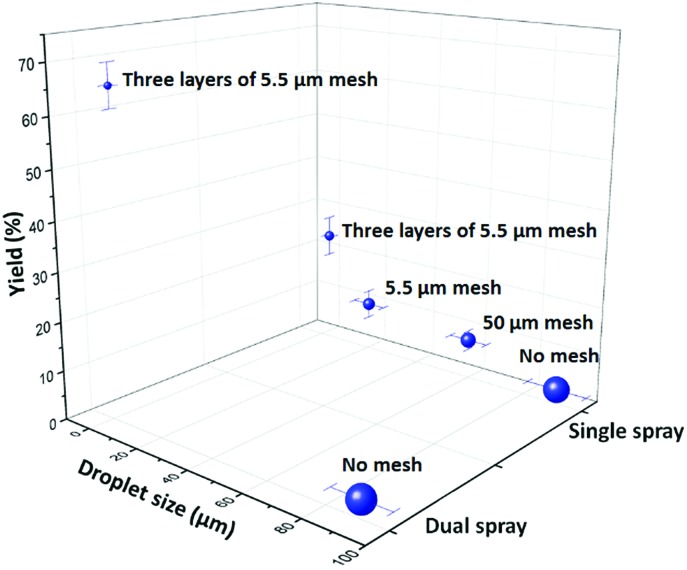
The microdroplet size distribution in a mixed solvent of water and ethanol (v/v = 1 : 1.2) generated by the internal-mix nozzle after mounting meshes with a size of 50 μm, 5.5 μm, and three layers of 5.5 μm which is plotted against the yield of 4-*tert*-butylbenzoic acid from oxidation of 4-*tert*-butylbenzaldehyde. Error bars on the droplet size represent one standard deviation (≥20 measurements). Error bars on the product yield represent one standard deviation (3 measurements).

We tried various methods to reduce the droplet size including using electrified droplet fission, and acceleration of droplet desolvation by heating the droplet flying path and extending the droplet flying distance (Fig. S6†). We found that the most efficient method was to mount meshes in front of the spray hole (Fig. 1b). Large droplets were broken into small droplets through size-guided Ni wire meshes. Scanning electron microscopy (SEM) images (Fig. 4) show meshes of 50 μm, 5.5 μm and three layers of 5.5 μm used in the study. PIV was used to measure the sizes of microdroplets generated by the internal-mix nozzle mounted with these meshes in a water–ethanol solution. The meshes effectively reduced the droplet sizes (Fig. 5), and by overlapping three layers of 5.5 μm mesh (the minimum size we purchased commercially), the droplet size was reduced to about 3 μm, which can be comparable to the size of microdroplets generated in the small sonic sprayer.

Another important factor that allows the reaction to have high yield is the mixing efficacy of gas and microdroplets. In order to increase the interactions between 4-*tert*-butylbenzaldehyde and O_2_, we introduced another stream of O_2_ through a similar nozzle but without infusing the liquid. The optimized angle between the two nozzles was set between 60° and 80°. Rapid mixing at the cross section of two fluid streams allows efficient mass transfer between the two phases. Finally, the aerobic oxidation of 4-*tert*-butylbenzaldehyde to 4-*tert*-butylbenzoic acid was achieved in a mixture of water and ethanol (v/v = 1 : 1.2) at a product formation rate of 10.5 mg min^–1^ with a yield of 66% for the pure product isolated by liquid chromatography. As Fig. 5 shows, the highest yield was obtained with small droplets in dual spray.

## Conclusions

In summary, we have demonstrated that aerobic oxidation can be carried out in microdroplets much more rapidly and with higher yield compared with their bulk-phase counterpart. Addition of catalytic nickel(ii) acetate further accelerated microdroplet reaction and increased the yield by about a factor of two. Aromatic, heterocyclic, and aliphatic aldehydes were oxidized to their corresponding carboxylic acids. O_2_ plays the dual role of being the sheath gas to generate microdroplets as well as the sole oxidant in the reaction. We also developed a high flux device for scaling up microdroplet synthesis using an internal-mix nozzle mounted with size-controlled meshes. We achieved a preparative synthesis of 4-*tert*-butylbenzoic acid at a rate of 10.5 mg min^–1^ with an isolated product yield of 66%, which demonstrates the possible practical utility of the present method.

## Conflicts of interest

There are no conflicts to declare.

## Supplementary Material

Supplementary information
