# Supplementary material for: Preparative microdroplet synthesis of carboxylic acids from aerobic oxidation of aldehydes
Source: Chem Sci. 2018 May 16;9(23):5207–11. doi: 10.1039/c8sc01580e (PMC6001248; doi:10.1039/c8sc01580e)

Electronic Supplementary Material (ESI) for Chemical Science.
This journal is © The Royal Society of Chemistry 2018

Supporting information

Preparative microdroplet synthesis of carboxylic acids from aerobic oxidation of aldehydes

Xin Yan, Yin-Hung Lai, and Richard N. Zare*

[*] X. Yan, Y. Lai, and R. N. Zare
Department of Chemistry, Stanford University
333 Campus Drive, Stanford, CA 94305-5080 (USA)
E-mail: zare@stanford.edu

1. General Experimental Details

1.1 Chemicals and materials

All chemicals were purchased from Sigma-Aldrich (St. Louis, MO) unless otherwise noted. Mesitylene was purchased from TCI (Purchasing-US@TCIchemicals.com). HPLC grade solvents were purchased from Fisher Scientific (Portland, OR). Spray nozzles were purchased from Unist Co., Grand Rapids, MI. Precision electroformed Ni meshes with sizes of 50 μm, and 8 μm (SEM shows 5.5 μm) were purchased from Precision Eforming LLC. (Cortland, NY). Capillaries were purchased from Polymicro Technologies. Parts to assemble sonic sprayer were ordered from IDEX Health & Science LLC and Swagelok. Syringes were purchased from Fisher Scientific.

1.2 Nuclear magnetic resonance (NMR) spectra were acquired on a Varian Mercury-400 operating at 400 MHz and 100 MHz, and are referenced internally to residual proton-containing solvent signals. $CDCl_3$ or D_2O was used as the solvent. 1,3,5-Trimethylbenzene was used as the internal standard for quantitation.

1.3 Gas chromatograph-mass spectrometry (GC-MS) spectrum was acquired on HP 7890/5975 GC-MS. The single quadrupole MS is equipped with an electron impact ionization (EI) source.

1.4 Scanning electron microscopy (SEM) analyses were performed on a Zeiss Sigma scanning electron microscope with a Schottky Field Emission (FE) source and a GEMINI electron optical column. A lateral Secondary Electron (SE) Detector was used. SEM analyses were operated at an accelerating voltage of 5 kV with a working distance of about 20 mm.

2. Microdroplet synthesis of carboxylic acids from aerobic oxidation of aldehydes

For the small-scale gas-liquid phase microdroplet synthesis of 4-*tert*-butylbenzoic acid **2**, 4-*tert*-butylbenzaldehyde (**1**, 0.1 M) and 5 mol% nickel(II) acetate in the water-ethanol mixed solvent (v:v = 1:1.2) were loaded into an airtight glass syringe. The solution was delivered with a syringe pump (Harvard Apparatus, Holliston, MA) at a flow rate of 15 mL/min to a capillary with an i.d. of 50 μm and o.d. of 360 μm. The end of the capillary was equipped with a sheath-gas-assisted spray emitter. Compressed O_2, which served as the sheath gas and oxidant, was operated at 90-120 psi. Optimized microdroplet trapping system (Fig. S1) was used to collect the plumes from the spray source. Upon completion of the reaction, dichloromethane was used to extract the product from water, and the product was dried by sodium sulfate.

For the preparative synthesis of 4-t*ert*-butylbenzoic acid, 4-*tert*-butylbenzaldehyde (**1**, 0.1 M) was dissolved in a water-ethanol mixed solvent (v:v = 1:1.2), and pumped through a pipeline from a tank pressurized by nitrogen gas (20 bar) to the spray nozzle. O_2 was operated at 60 psi, and split into two streams: one was introduced to the nozzle housing to mix with the liquid of 4-*tert*-butylbenzaldehyde and disperse it into microdroplets; the other was supplied for further mixing. Large column (i.d. 15 cm) with a sand core was used to collect the product. Upon completion of the reaction, the product was extracted with dichloromethane, dried by sodium sulfate, and purified by column chromatography with acetyl acetate and hexane (v:v = 1:3).

3. Droplet Size Measurement

Micro-particle image velocimetry (μPIV) was used to measure the droplet sizes in the study. The method is similar to that reported for imaging the electrospray plume (E. T. Jansson, Y.-H. Lai, J. G. Santiago, R. N. Zare, *J. Am. Chem. Soc.* **2017**, *139*, 6851-6854). Briefly, the determination of droplet sizes was done by elastic light scattering using pulsed 2nd harmonic Nd:YAG lasers (λ= 532 nm) plus additional optics. An objective (5x magnification, NA= 0.15) was used to gather light and produce imaging onto an interline-transfer CCD camera with a double-frames imaging feature. In this method, the imaging recorded by CCD is the convolution of the point response function, which depends on the optics and illumination wavelength, and the actual droplet size. The droplet size is calculated based on the average number of pixels that droplets occupy on the imaging plane. The surface-area-to-volume (SA/V) ratio of microdroplets is dervied from the droplet size. It should be noticed that droplets with a size less than 1.3 μm in diameter will be recognized as having a size of about 1.3 μm owing to the point response function.

4. Microdroplet trapping system

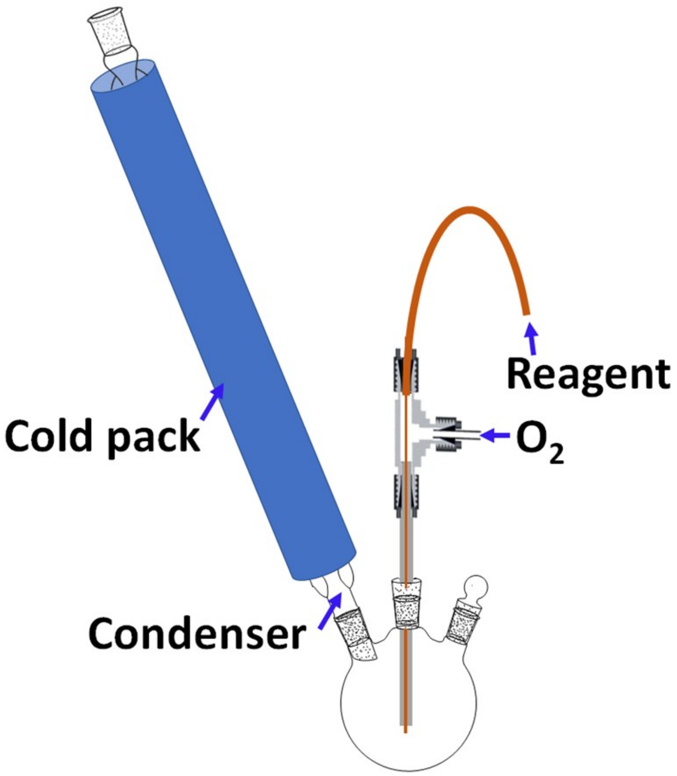

Fig. S1 Trapping system for microdroplet reactions.

5. **Screening of catalysts for oxidation of 4-*tert*-butylbenzaldehyde in microdroplets**

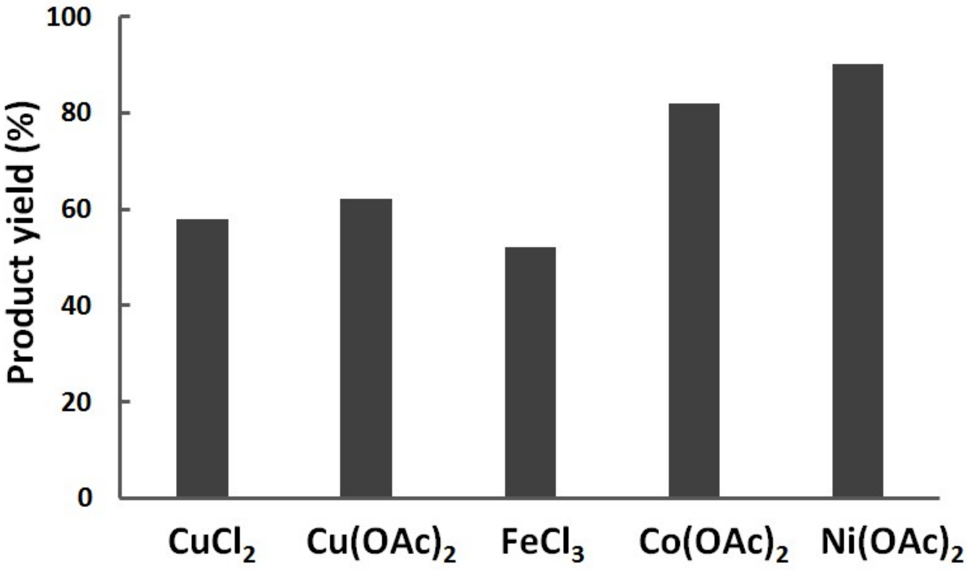

Fig. S2 Oxidation yield of 4-*tert*-butylbenzoic acid from 4-*tert*-butylbenzaldehyde with molecular oxygen in water-ethanol (v:v = 1:1.2) in microdroplets catalyzed by 5 mol% $CuCl_2$, $Cu(OAc)_2$, $FeCl_3$, $Co(OAc)_2$, or $Ni(OAc)_2$.

6. Oxidation of 4-*tert*-butylbenzaldehyde in bulk

We monitored the bulk oxidation of 4-*tert*-butylbenzaldehyde in a water-ethanol solvent (v:v = 1:1.2) with molecular oxygen as a control experiment for microdroplet reaction. The bulk reaction was vigorously stirring at room temperature. Without the addition of nickel(II) acetate, less than 1% of product was detected after 30 min. With the addition of 5%mol of catalytic nickel(II) acetate, the product 4-*tert*-butylbenzoic acid was formed in a yield of 4% after 30 min. We continuously monitored the bulk reaction for 24 hours and 6% of product was detected. Possible reasons for the slow kinetics in bulk are solvent effect (Angew. Chem. Int. Ed. 2008, 47, 2849-2852.; RSC Adv., 2013, 3, 18931-18937.), as well as the instability of the catalyst in the absent of ligands and additives. Because this work focuses on the microdroplet synthesis, we optimized the solvent (Fig. S8) for microdroplet reactions but not in bulk. In addition, microdroplet reactions commonly happen in milliseconds, which may overcome the instability of the catalyst without ligands.

7. The effect of the surface-area-to-volume (SA/V) ratio of microdroplets on the yield of the aerobic oxidation

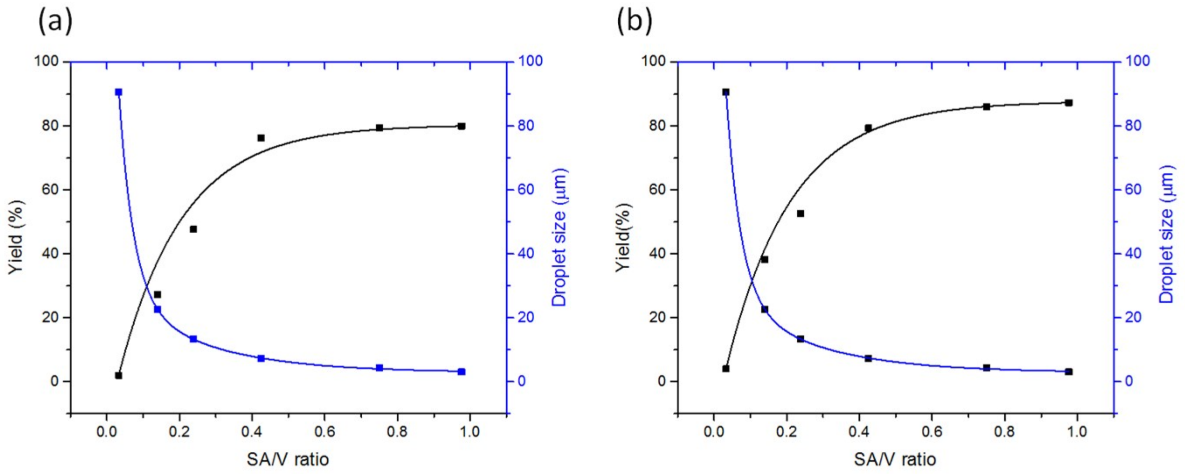

Fig. S3 Dependence of product yield on SA/V ratio in microdroplet aerobic oxidation of (a) benzaldehyde and (b) 2,4,6-trimethylbenzaldehyde.

8. **Screening of solvents for oxidation of 4-*tert*-butylbenzaldehyde without the present of catalysts in microdroplets**

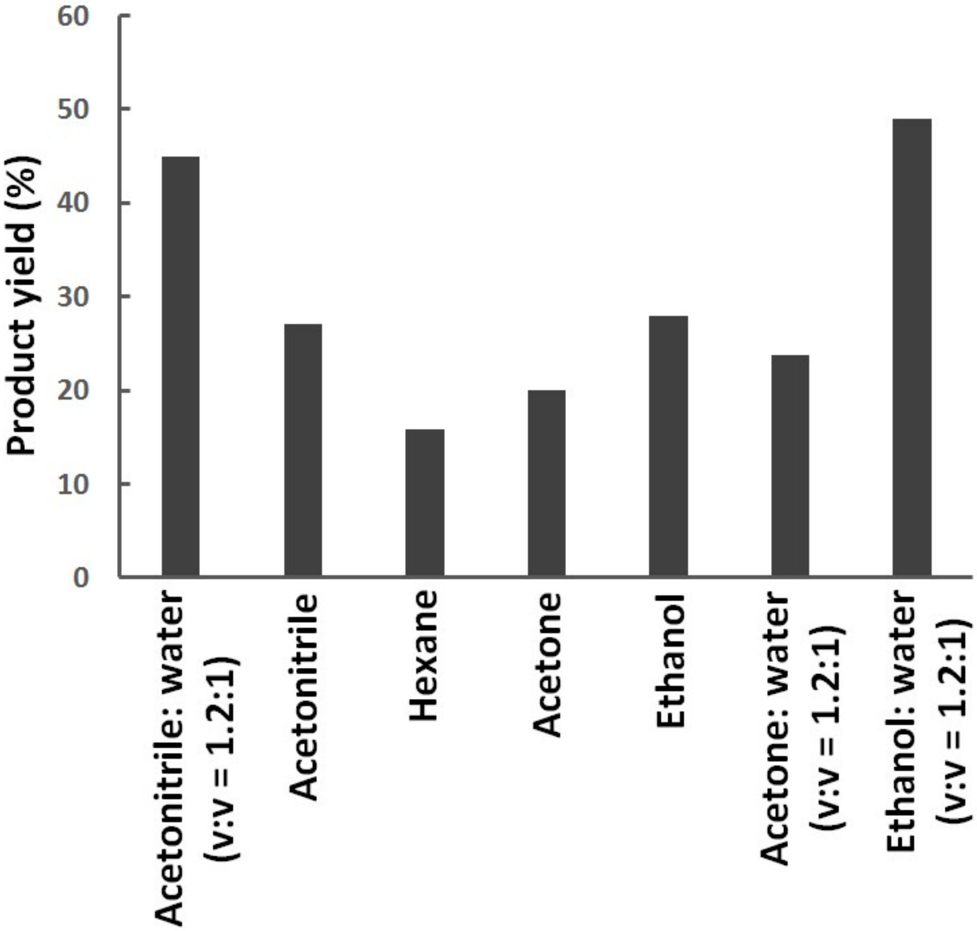

Fig. S4 Oxidation yield of 4-*tert*-butylbenzoic acid from 4-*tert*-butylbenzaldehyde with molecular oxygen in microdroplets in acetonitrile: water (v:v = 1.2:1), acetonitrile, hexane, acetone, ethanol, acetone: water (v:v = 1.2:1), or ethanol: water (v:v = 1.2:1). Note: Aqueous solution shows the better yields, while the low solubility of 4-*tert*-butylbenzaldehyde in pure water limits its use as the solvent in the microdroplet reaction.

9. Devices for large-scale microdroplet synthesis

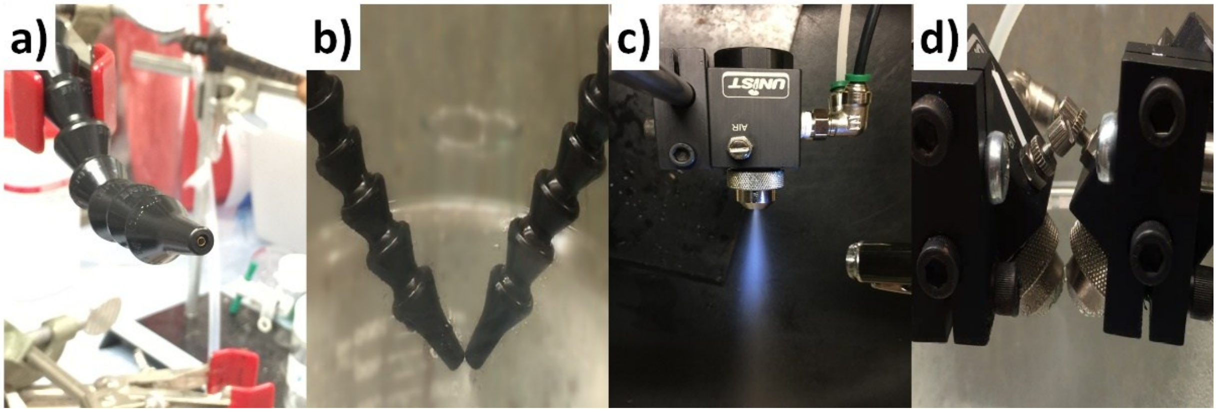

Fig. S5 a) Large single external-mix spray nozzle, b) dual external-mix spray nozzle, c) single internal-mix spray nozzle without mesh d) dual internal-mix spray nozzle with three layers of 5.5 μm mesh.

10. Methods of reducing the droplet size besides using size-control meshes

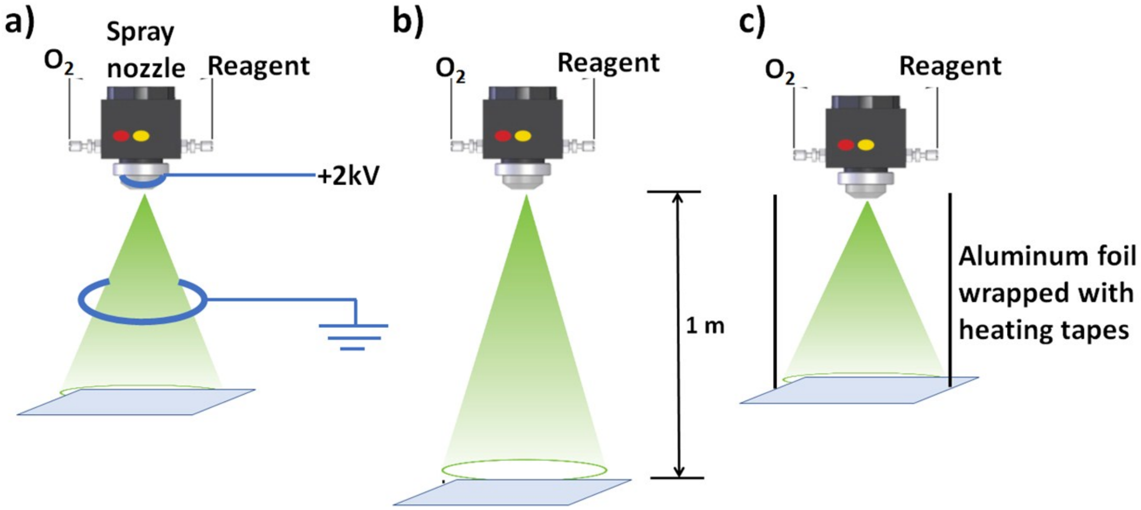

Fig. S6 Methods of reducing the droplet size: a) using electrified droplet fission, and acceleration of droplet desolvation by b) extending droplet flying distance, and c) heating the droplet flying path

11. ^{1}H NMR and GC-MS spectra of products (carboxylic acids) from microdroplet aerobic oxidation of aldehydes

11.1 4-*tert*-Butylbenzoic acid

^{1}H NMR (400 MHz, Chloroform-*d*) δ 8.10 – 8.01 (m, 2H), 7.54 – 7.46 (m, 2H), 1.35 (s, 9H).

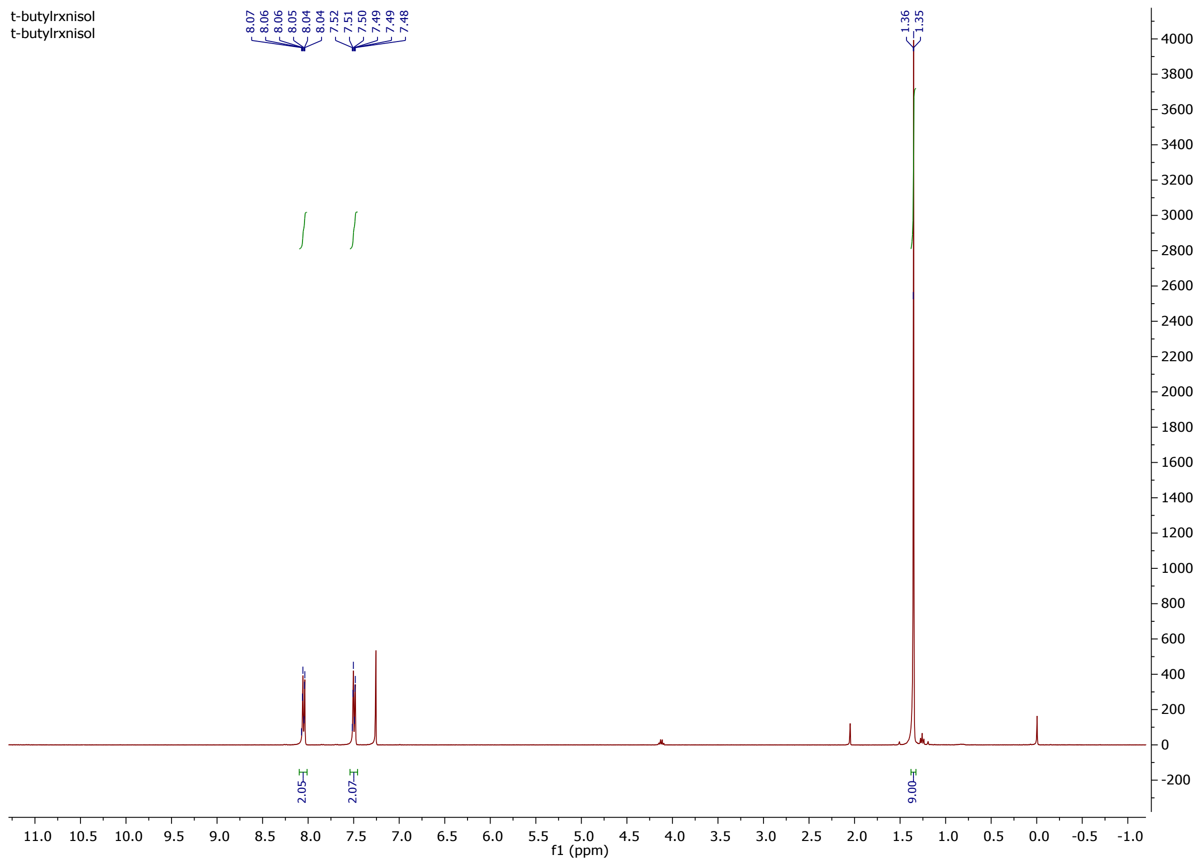

11.2 Cyclohexanecarboxylic acid

^{1}H NMR (400 MHz, Chloroform-*d*) δ 2.33 (td, *J* = 11.1, 3.1 Hz, 1H), 1.97 – 1.89 (m, 2H), 1.81 – 1.72 (m, 2H), 1.65 (d, *J* = 10.1 Hz, 1H), 1.44 (tt, *J* = 12.3, 6.9 Hz, 2H), 1.27 (tt, *J* = 19.1, 10.4 Hz, 3H).

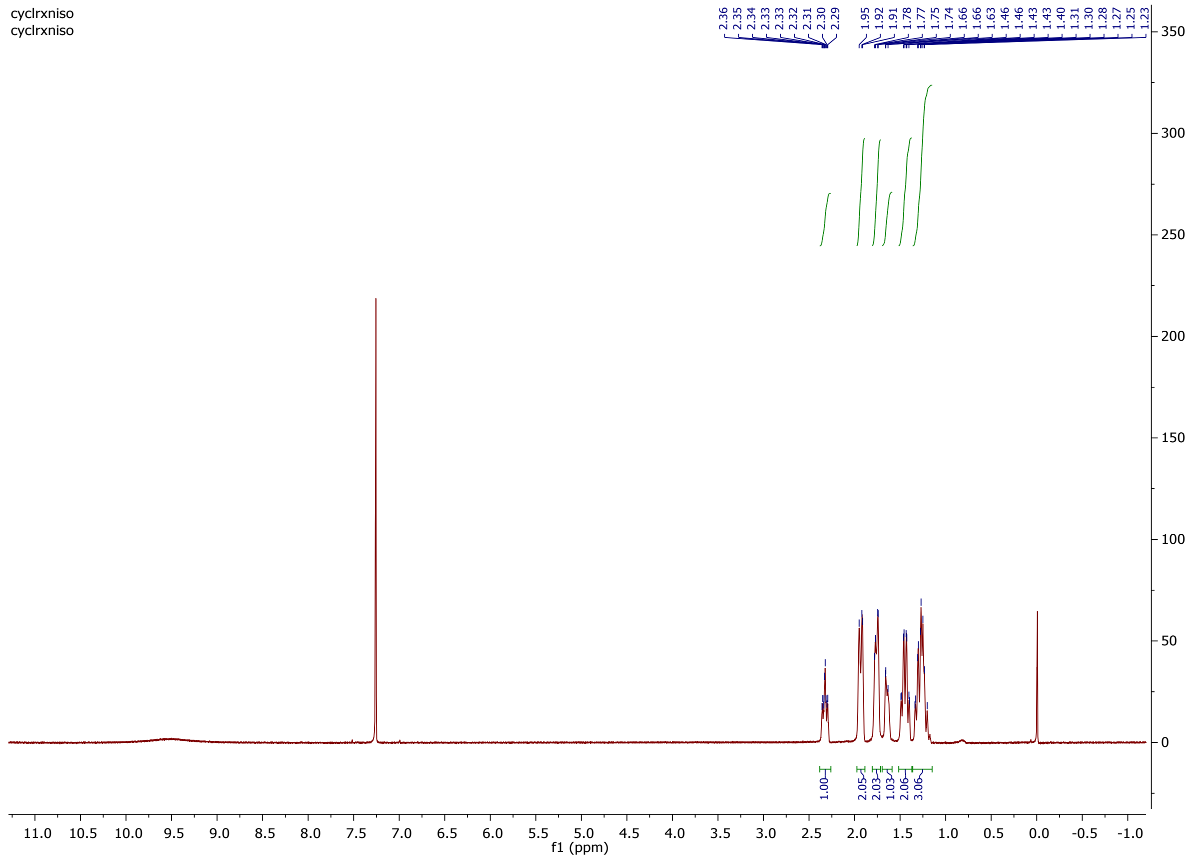

8.3 1-Naphthoic acid

^{1}H NMR (400 MHz, Chloroform-*d*) δ 9.10 (d, *J* = 8.7 Hz, 1H), 8.47 – 8.39 (m, 1H), 8.11 (d, *J* = 8.1 Hz, 1H), 7.93 (d, *J* = 8.2 Hz, 1H), 7.72 – 7.63 (m, 1H), 7.62 – 7.52 (m, 2H).

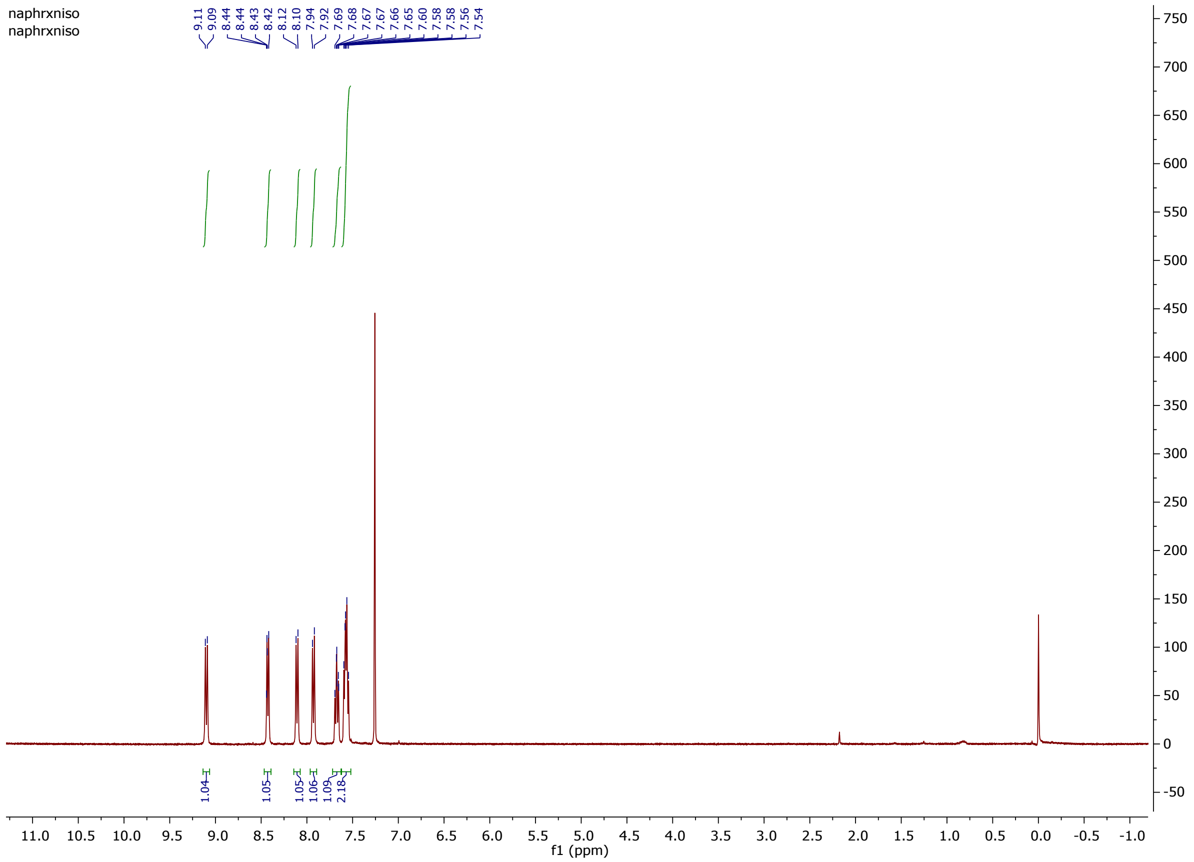

8.4 Benzoic acid

^{1}H NMR (400 MHz, Chloroform-*d*) δ 8.18 – 8.09 (m, 2H), 7.62 (ddt, *J* = 7.6, 6.6, 1.1 Hz, 1H), 7.49 (t, *J* = 7.7 Hz, 2H).

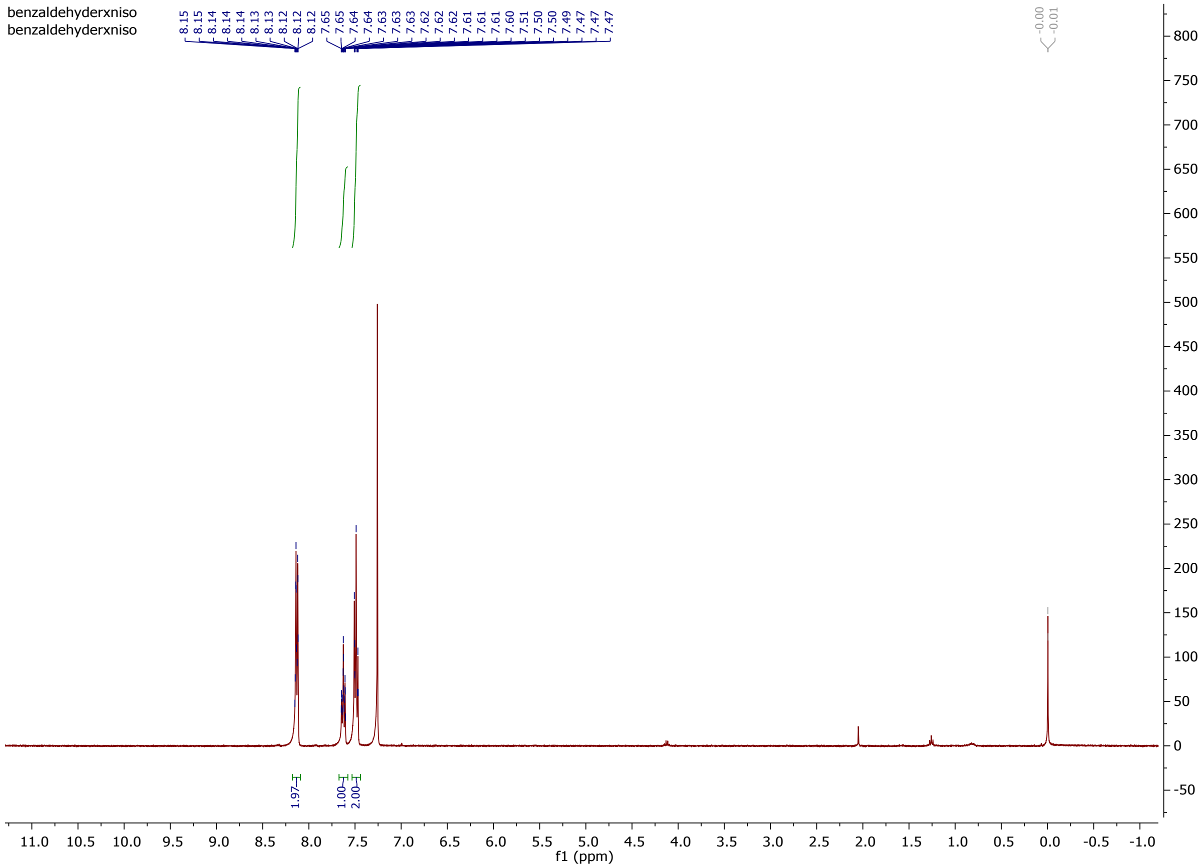

8.5 2,4,6-Trimethylbenzoic acid

^{1}H NMR (400 MHz, Chloroform-*d*) δ 6.88 (s, 2H), 2.40 (s, 6H), 2.29 (s, 3H).

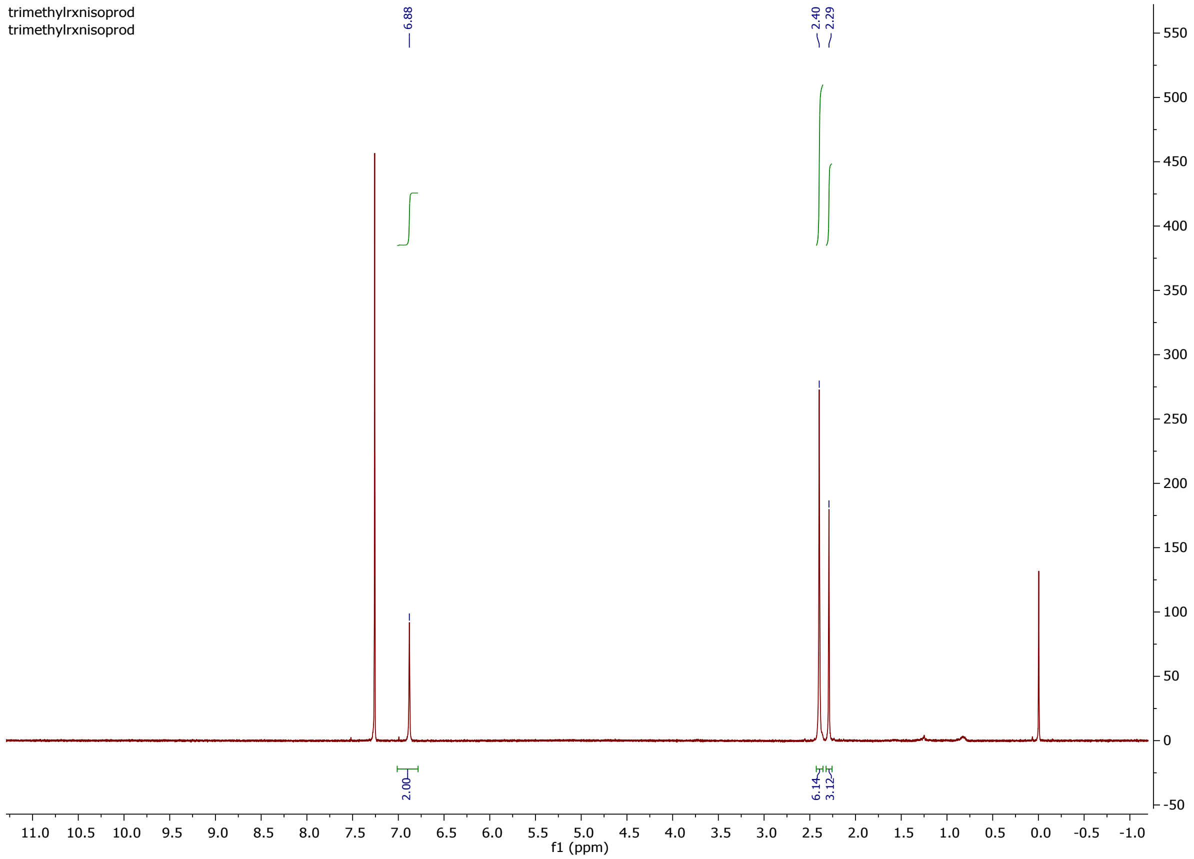

8.6 Nicotinic acid

^{1}H NMR (400 MHz, Deuterium Oxide) δ 8.91 (s, 1H), 8.69 (dq, *J* = 8.1, 1.7 Hz, 1H), 8.62 (dq, *J* = 5.8, 1.0 Hz, 1H), 7.92 – 7.83 (m, 1H).

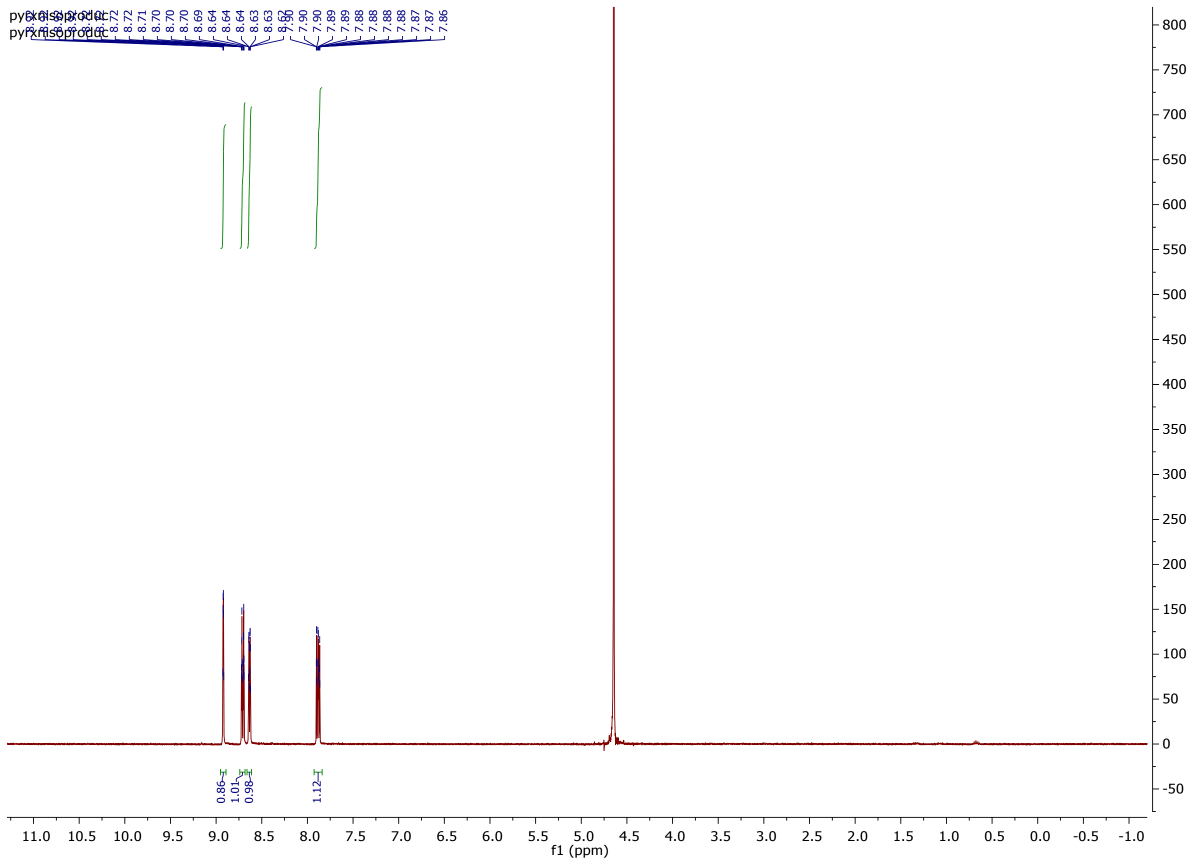

8.7 2-Ethylhexanoic acid

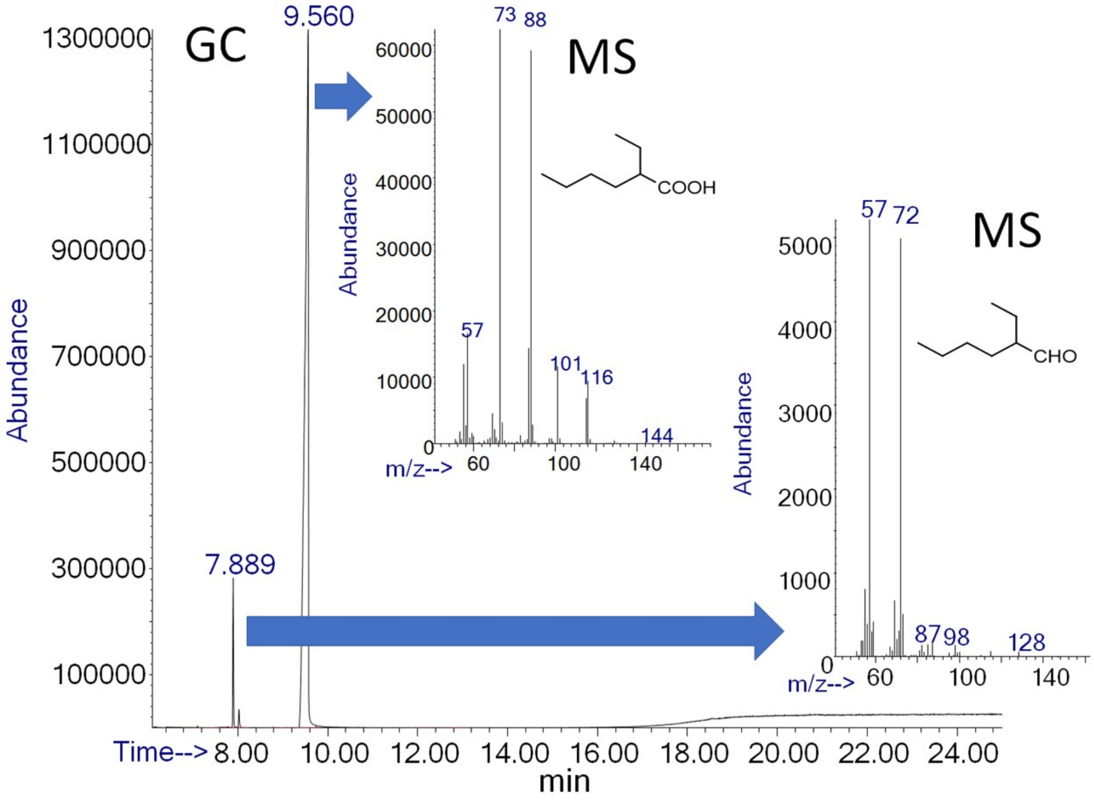

Supplement: Supplementary file 1 [file SC-009-C8SC01580E-s001.pdf]
